# Suicide prevention for substance using youth experiencing homelessness: study protocol for a randomized controlled trial

**DOI:** 10.1186/s13063-024-07997-y

**Published:** 2024-03-09

**Authors:** Natasha Slesnick, Brittany Brakenhoff, Laura J. Chavez, Caleb L. Cuthbertson, Ruri Famelia, Xin Feng, Jodi Ford, Eugene Holowacz, Soren Jaderlund, Kelly Kelleher, Ellison Luthy, Allen M. Mallory, Alexis Pizzulo, Tatiana D. Slesnick, Tansel Yilmazer

**Affiliations:** 1 Department of Human Sciences, College of Education and Human Ecology, The Ohio State University, 1787 Neil Ave, Columbus, OH 43210 USA; 2 Center for Child Health Equity and Outcomes Research, Nationwide Children’s Hospital, 700 Children’s Drive, Columbus, OH 43205 USA; 3 College of Nursing, The Ohio State University, 1577 Neil Ave, Columbus, OH 43210 USA; 4 College of Food, Agriculture and Environmental Science, School of Environment and National Resources, 2021 Fyffe Rd, Columbus, OH 43210 USA; 5 Department of Pediatrics, College of Medicine, The Ohio State University, 370 W 9th Avenue, Columbus, OH 43210 USA

**Keywords:** Youth homelessness, Suicide prevention, Substance use, Randomized controlled trial

## Abstract

**Background:**

While research on substance using youth experiencing homelessness (YEH) is increasing, there is a dearth of information regarding effective prevention interventions for these youth. Suicide is the leading cause of death among YEH and most youth do not access services that may be available to them. Therefore, this study seeks to address this gap in the research literature with the goal to identify an effective suicide prevention intervention that can be readily adopted by communities that serve these youth.

**Methods:**

Three hundred (*N* = 300) YEH with recent substance use and suicidal ideation or a recent suicide attempt will be recruited from the streets as well as a drop-in center serving YEH. After the baseline assessment, all youth will be randomly assigned to Cognitive Therapy for Suicide Prevention (CTSP) + Services as Usual (SAU) (*N* = 150) or to SAU alone (*N* = 150). SAU includes outreach, advocacy, and service linkage whereas YEH who receive CTSP will also receive ten CTSP sessions and an optional nine booster sessions. Follow-up assessments will be conducted at 3, 6, 9, and 12 months post-baseline. Theoretically derived mediators (e.g., cognitive distortions) will be tested to shed light on mechanisms associated with change, and the moderating effects of sex, race, sexual orientation, and baseline service connection will be examined. In order to ease future dissemination of the intervention to agencies serving YEH, we will rigorously assess acceptability, feasibility, fidelity, and cost associated with the delivery of our intervention approach using a mixed-methods approach.

**Discussion:**

This study adds to a very small number of clinical trials seeking to prevent lethal suicide among a very high-risk group by addressing suicidal ideation directly rather than underlying conditions. It is hypothesized that youth receiving CTSP + SAU will show greater reductions in suicidal ideation (primary outcome), substance use, and depressive symptoms (secondary outcomes) over time compared to SAU alone, as well as improved risk and protective factors.

**Trial registration:**

NCT05994612. Date of Registration: August 16, 2023.

## Introduction

### Background and rationale {6a}

Homelessness is associated with a range of poor health outcomes including exacerbation or initiation of mental and physical health problems, victimization, premature mortality, and substance use. In fact, up to 95% of YEH report recent substance use [1]. Of additional concern are the extremely high rates of attempted and completed suicide [2]. Suicide is the leading cause of death among YEH, with national studies indicating that up to 68% of samples of these youth report having at least one lifetime suicide attempt [3]. Replicable, evidence-based suicide preventive interventions for youth, in general, are limited and are non-existent for YEH.

This randomized trial seeks to extend prior research by emphasizing engagement in treatment and increasing the reach of CTSP by also engaging youth recruited from the streets who are not connected to community services. Unfortunately, the majority of intervention studies have excluded YEH who were not already engaged in services through drop-in centers or other shelter services, limiting our knowledge of them and our ability to effectively intervene. Yet, one study indicated that only 10% of YEH access community resources meant to serve them [4].

#### YEH and suicidal risk

Overall, studies estimate that between 20 and 68% of YEH have attempted suicide at least once in their lifetime [5, 6]. This rate is much higher than that observed in the general youth population, for example, the Centers for Disease Control [7] found that 8.9% of youth report a prior suicidal attempt. Among YEH who have attempted suicide, an average of 6.2 attempts is reported. In addition, lifetime suicidal ideation rates have ranged from 14 to 66.5% [8]. Given that suicidal ideation is a robust predictor of suicide attempts, and suicide attempts are likely to be infrequent in the 12-month study period, suicidal ideation was chosen as our primary targeted outcome. However, suicide attempts will also be assessed.

YEH experience trauma both before and after becoming homeless [9] which contribute to suicidal thoughts and attempts [10]. Rates of physical and sexual abuse range between 16 and 60% [11]. Rew et al. [12] found that YEH who had experienced physical or sexual abuse were 1.8–3 times more likely to attempt suicide compared to YEH without abuse histories. Sexual abuse may be particularly influential on suicidal thoughts and behaviors. For example, Kidd [10] found that the impact of physical abuse on suicidal behavior faded once youth left home, while sexual abuse continued to predict suicidality even after youth left their homes. Overall, youth who have experienced childhood abuse are at an increased risk of suicide. However, experiences on the street can also contribute to suicidal behavior among YEH. In particular, sexual victimization (e.g., rape) has been associated with higher rates of suicidal ideation and attempts [8]. Other risks include street victimization, sex trafficking, criminal involvement, and substance abuse [13]. In sum, the lives of YEH are often characterized by violence, chaos, abuse, and neglect prior to and after becoming homeless, which itself has been associated with higher suicide risk.

High rates of substance use, between 70 and 95%, are consistently reported among YEH [1] and studies report that 60–71% of YEH meet diagnostic criteria for a substance use disorder [14]. Therefore, substance use is considered the norm rather than the exception among YEH. Some research suggests that substance use predicts suicidal ideation and attempts among YEH [6, 10, 15]. In Kidd [16] qualitative interviews with 80 YEH, many youth discussed how their drug use led to the experience of feeling trapped, which made them feel that suicide was the only way to end their suffering. However, suicidal thoughts and depressive symptoms can also increase risk of substance use as one study showed that many youth acknowledged that problem substance use was a slow and passive form of suicide in which they gave up on life [16]. History of depression is a consistent predictor of death by suicide and attempts among youth as well [17]. Depression is high among YEH, with rates of clinical depression ranging from 29 to 83.6% [8]. Therefore, substance use and depressive symptoms are secondary outcomes in this study. The link between completed or attempted suicide, suicidal ideation, substance use, and depressive symptoms has been documented, yet much work remains to identify the mechanisms underlying this association.

#### Risk and protective factors underlying suicidal ideation (mediators)

General cognitive theory (GCT) asserts that perceptions shape emotions which drive behavior [18]. Rudd [19] and Joiner et al. [20] expanded GCT for understanding suicidal behavior. In his model, Rudd includes processes that bring about psychiatric disturbances, activate negative schemas, and exacerbate distress in suicidal individuals. According to Rudd’s theory, the more suicide-specific risk factors a person has, the more likely it is that the person’s negative suicidal schema will be activated. Another psychological theory for understanding suicidal acts was developed by Joiner [21] and postulates that individuals must have two maladaptive cognitive states for a suicide attempt to occur, low belongingness and perceived burdensomeness, as well as the ability to enact self-injury. Perceived burdensomeness is the belief that an individual “burdens family, friends, and/or society” and that the individual has more value deceased than living [36, p.634]. Low belongingness is the perception that an individual is socially isolated from family members, friends, or other groups [20]. While feelings of burdensomeness and low belonging may instill a desire to terminate life, according to this theory, they are insufficient to warrant a suicidal act. Repeated exposure to painful or otherwise provocative events (habituating events), such as sexual abuse, physical abuse, and self-harm diminish individuals’ natural self-preservation instinct and fear of death, thus increasing one’s ability to complete suicide. For example, painful events might lead to suicide attempts, and past suicidal attempts can predict future lethal suicide, increasing ability to enact self-injury [21, 22]. This integrated model, using cognitive and habituation concepts, was first proposed by Wenzel et al. [23] and has been used successfully to guide suicide interventions, such as CTSP, with adults and adolescents. The model expands on Beck’s general cognitive theory by integrating cognitive models that explain underlying suicide-specific processes.

**Fig. 1:**
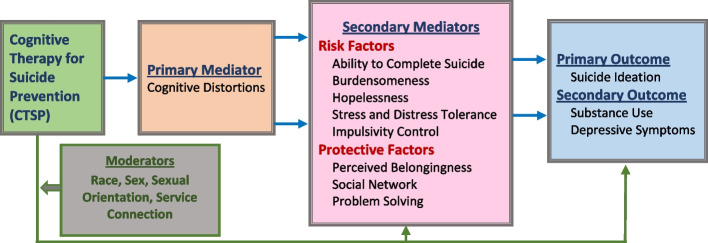
Conceptual model

Social connections with family and friends have an impact on factors associated with suicidality among YEH. Youth from abusive families are less likely to perceive support from their family members, and thus turn to friends and peers for emotional support [24]. Contact with peers has been shown to have positive and negative effects on YEH’s suicidal behavior [25]. Peers on the streets may be a source of information, mentoring, and support, as well as victimization and/or coercion. Affiliation with pro-social peers predicts lower levels of psychological distress [26] and depression [24]. Relationships with deviant peers are a risk factor for depression, substance use, and suicidal behavior [27] as is social withdrawal [6]. Relatedly, social problem-solving is a specific type of problem-solving ability that has been linked to suicidal ideation. The term refers to problem-solving as it occurs in everyday life and is defined as a “complex, cognitive-affective-behavioral process” (p. 156) by which a person attempts to develop relevant ways of coping with stressful situations [28]. A great deal of research has provided evidence for the link between poor problem-solving skills and suicidality. Effective problem-solving has been associated with low scores on measures of hopelessness as well as with suicidal ideation [29]. In contrast, cognitions and emotions that impede effective problem-solving have been found to be positively associated with hopelessness and suicidal ideation [30]. Please see Fig. 1, our conceptual model, below.

#### Moderating variables

Several variables may be associated with intervention outcomes and will be examined as moderators in statistical analyses (race, sexual orientation, sex, and baseline service connection). The relationship between sex and increased risk for suicide attempts has been found to be consistent among YEH with female youth attempting suicide more often than males [8, 10]. However, females complete suicide less often than males [31]. Youth who identify as Black and/or as a sexual minority are overrepresented in the YEH population. Compared to YEH who identify as White, Black YEH have decreased risk of suicidal thoughts [32] and attempts [33]. However, sexual minority youth are more than twice as likely to attempt suicide than their heterosexual counterparts [7]. Finally, some research suggests that service-connected youth have different needs than service disconnected youth. Service disconnected youth report more severe mental health problems, substance use, and other risks [34]. Therefore, youth not accessing services are likely the most in need of assistance, and the least likely to receive it, which is of concern since service connection is a consistent predictor of improved mental health, biological, and social functioning overall [26]. An examination of individual variation through the moderation analyses will allow further specification within subgroups of treatment response.

### Objectives {7}

In summary, this study focuses on reducing suicidal ideation among YEH who are underserved by the services system. Through this study, we will test our strategy for significantly reducing suicidal ideation (primary outcome), substance use, and depressive symptoms (secondary outcomes) as well as reducing risk factors and strengthening protective factors. Our intervention and conceptual model are based upon Beck’s general cognitive theory [18]. Finally, we will rigorously assess acceptability, feasibility, fidelity, and cost to guide future dissemination efforts. Study aims and hypotheses are listed below:

- *Specific Aim 1*. Test the effects of outreach-worker delivered CTSP + SAU versus SAU alone on suicidal ideation (primary outcome), substance use, and depressive symptoms (secondary outcomes) at 3, 6, 9, and 12 months.
- *Hypothesis*. Youth assigned to CTSP + SAU will show statistically significant improvement on the study outcomes over time compared to those assigned to SAU alone.
- *Specific Aim 2*. Test the mediating relationship between the intervention and primary and secondary outcomes.
- *Hypothesis 2a*. Cognitive distortions will mediate the treatment effects on outcomes.
- *Hypothesis 2b*. The relation between cognitive distortions and outcomes will be further mediated by the risk and protective factors. That is, lower cognitive distortions at the 3-month follow-up will be related to higher levels of protective factors and lower levels of risk factors at 6 months, which in turn will be related to reduced suicidal ideation, substance use, and depressive symptoms at the 12-month follow-up.
- *Specific Aim 3*. Explore how moderators (baseline service connection, sex, race, sexual orientation) affect individual’s response to CTSP.
- *Specific Aim 4*. Rigorously assess acceptability, feasibility, fidelity, and cost of outreach-worker delivered CTSP using a mixed-methods approach for future adoption studies.

### Trial design {8}

**Fig. 2:**
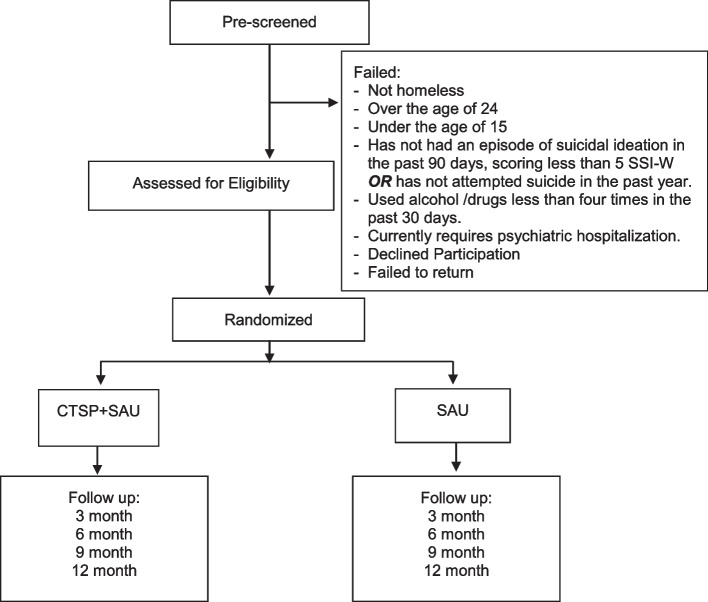
Consort flow chart

The study is designed as a randomized controlled, parallel group, two-arm, superiority trial. All youth (*N* = 300) will be randomly assigned to the cognitive therapy for suicide prevention (CTSP) + SAU (*n* = 150) or to SAU alone (*n* = 150). SAU includes outreach, advocacy, and service linkage and is the standard intervention among providers serving populations experiencing homelessness [35]. The design of this study will allow us to test the intervention as an add-on to standard community-based services (SAU) so that the unique needs of these underserved youth are addressed in a holistic fashion. Advocates will be embedded in condition in order to reduce potential contamination. That is, only advocates trained in CTSP will deliver CTSP + SAU. And finally, as the SAU mirrors that used by shelters and drop-in centers, if the intervention shows significant effects above and beyond SAU, evidence for disseminating the intervention to drop-in centers around the country will be provided. As the intervention is manualized and uses cognitive behavioral strategies, portability is enhanced. SAU and CTSP intervention meetings must be completed by 6 months post-baseline. Self-report questionnaires and interviews will be conducted at baseline and at 3, 6, 9, and 12 months post-baseline. An intent to treat design will be followed so that all youth, regardless of participation in the intervention conditions, will be tracked for follow-up. Please see Fig. 2 below.

## Methods: participants, interventions, and outcomes

### Study setting {9}

This is a single-site study conducted at The Ohio State University, USA. The setting includes a drop-in center for YEH.

### Eligibility criteria {10}

1. Youth are between the ages of 15 to 24 years. Homeless youth is a term commonly used to describe homeless adolescents and young adults up to the age of 24 [9].
2. Youth reports at least one episode of suicidal ideation in the past 90 days, scoring 5 or higher on the Scale for Suicide Ideation – Worst Point (SSI-W). Severe suicidal ideation is defined as scoring 16 or higher on SSI-W. Beck et al. [36] reported that clients who scored 16 or higher on the SSI-W had 14 times higher chance to die by suicide. Given our prevention goal, we will engage those with a score of 5 or higher so that moderate risk youth are engaged as well. Similar to a study by Stanley and colleagues [37], suicidal ideation will be considered recent if it occurred in the prior 90 days; OR
3. Any self-reported suicide attempt in the past year.
4. Youth meets criteria for homelessness as defined by the McKinney-Vento Act as those who lack a fixed, regular, and adequate nighttime residence; lives in a welfare hotel, or place without regular sleeping accommodations; or lives in a shared residence with other persons due to the loss of one’s housing or economic hardship. This definition of homelessness is inclusive and is meant to capture the multiple locations where YEH seek refuge.
5. Youth reports at least *four* uses of alcohol/drugs in prior 30 days. Previous studies with youth indicate that using alcohol and/or illicit drugs more than three times per month is more characteristic of a problematic substance use pattern than general experimentation [38].
6. Youth does not require psychiatric hospitalization. However, if hospitalized, youth will be eligible for the study once released from the hospital.

### Who will take informed consent? {26a}

All youth will be recruited from a local drop-in center, as well as through outreach efforts at places like sandwich lines, soup kitchens, libraries, and on the streets. Research assistants (RAs) will approach youth to assess their interest in participating in the project. If a youth is interested, they will be interviewed by an RA who will use basic eligibility criteria as a guide. The consent or assent form and details of project participation will be reviewed in a private and comfortable location, primarily the research offices at the drop-in center. RAs will obtain written consent or assent from research participants. Those who meet the eligibility criteria will be scheduled for a baseline/intake evaluation. Youth found to be ineligible will receive referrals to area service providers.

### Additional consent provisions for collection and use of participant data and biological specimens {26b}

This trial collects hair samples in order to assay cortisol levels. No additional consent provisions are provided.

## Interventions

### Explanation for the choice of comparators {6b}

The comparator condition is services as usual (SAU), in which youth are linked to services within the community. SAU includes strengths-based outreach and advocacy which are typical services offered by agencies serving YEH. Drop-in centers typically also provide basic services, crisis counselors, and case managers onsite.

### Intervention description {11a}

#### Services as usual (SAU)

Services as usual (SAU) includes standard suicidal ideation practice. Outreach, engagement, and service linkage is standard intervention for finding and engaging those experiencing homelessness into services [35]. The goal of the outreach worker is to engage homeless youth through non-office contact in sandwich lines/soup kitchens, homeless camps, libraries, and parks and encourage youth to accept the next level of service. We refer to outreach workers as advocates, because they do more than outreach. Once an individual is engaged in the current project, the outreach worker/advocate will continue to work with the client for 6 months, helping youth to secure needed services and remaining a support for the youth as he/she traverses the system of care. The approach used here, Strengths-based Outreach and Advocacy (SBOA), follows the Strengths Model [39] which focuses on client strengths rather than deficits and ensures that the youth directs the services they receive.

#### Cognitive Therapy for Suicide Prevention (CTSP) + SAU

In the experimental condition, youth will receive SAU in addition to CTSP [23]. CTSP asserts that maladaptive cognitions lead to maladaptive emotional and behavioral responses. Furthermore, suicidal behaviors rather than underlying conditions are the primary focus. Therefore, CTSP seeks to identify triggers for suicidal behaviors, primarily targeting those cognitions associated with core beliefs and automatic negative thoughts. Therapists help youth replace negative cognitions with alternative, more adaptive ones which are expected to lead to positive behavioral change. CTSP is offered in ten sessions, but, similar to Stanley et al. [37] and Brent et al. [40], youth will be offered up to nine additional maintenance sessions within the first 6 months post-baseline. A crisis plan is developed in the first session and is expanded as therapy progresses. It includes emergency contact numbers as well as positive coping behaviors, such as walking or listening to music that the client can perform alone. Consistent with the Interpersonal Theory of Suicide [41] and Rudd’s theory [19], the experience of prior suicidal behaviors is considered associated with increased risk for future suicide attempts. Therefore, assessment of recent suicidal thoughts and behaviors is conducted every session. Clients in need of additional mental health treatment or services are referred to those respective agencies.

#### Training and supervision

Training in CTSP will only be offered to those who will deliver CTSP in order to prevent contamination. Training consists of readings (manual/book) [23], a 3-day training in the intervention, including role play exercises and ongoing bi-weekly supervision. Master’s-level providers will be hired from the field with experience working with homeless populations and a comfort with nontraditional settings. Providers of CTSP + SAU and SAU will also participate in a 2-day training by the PI (Slesnick) on engagement, tracking, and service linkage. Training will include in vivo observation and modeling by PI Slesnick to ensure they are comfortable identifying and engaging youth. Readiness for conducting the interventions independently will be determined by the advocate in consultation with the PI.

#### Acceptability, feasibility, fidelity, and cost (Aim 4)

Procedures for assessing acceptability, feasibility, fidelity, and cost of CTSP include both qualitative interviews and process measures as described below.

##### Qualitative interviews

First, qualitative interviews will be used to increase our understanding of acceptability and feasibility of CTSP among YEH. Up to 20 youth (or until saturation is reached) who received CTSP will be interviewed after completing the CTSP treatment period (6 months). Interviews will follow a structured interview guide that will elicit youth perspectives regarding their experience with the treatment, specifically what they feel they benefited from and what they did not find helpful. This will allow for a better understanding of factors that promote or impede engagement. We will also interview outreach workers/advocates who delivered CTSP and community stakeholders (*n* = 5). Community stakeholders will include representatives from the drop-in center and other local agency providers. Interviews will include open-ended questions about what it takes to implement CTSP, what could get in the way, and what could help. In addition, specific probes will be included that focus on domains from a widely used implementation framework, the Consolidated Framework for Implementation Research (CFIR) [42].

##### Process measures

Process measures are based on the RE-AIM framework [43] and implementation research definitions of outcome constructs [44]. Study records will be used to capture CTSP treatment acceptability including (1) number of sessions attended, (2) proportion completing high levels of therapy (80%), Client Satisfaction Questionnaire total scores (CSQ) [45], and (3) the Working Alliance Inventory scores (WAI) [46]. CTSP *fidelity* will be assessed with the (CTRS), an 11-item scale developed to assess outreach worker competence. Items are scored on a 7-point scale from 0 (Poor) to 6 (Excellent). The 11 items are summed to yield a CTRS total score, ranging from 0 to 66 (≥ 40 considered competent). The CTRS will be completed by the study’s PI through review of the digital session recording. CTSP *costs* will be measured as the outreach worker time for training, delivering CTSP, supervision, and contacts with youth for follow-up appointments. Costs will include the labor, equipment, and space costs, based on an activity-based costing method [47]. The instrument to collect and organize the cost of CTSP has been created by Co-I Yilmazer [48].

### Criteria for discontinuing or modifying allocated intervention {11b}

If the therapeutic is found to cause harm to participants (e.g., increase in suicidal ideation or substance use), the trial will be stopped.

### Strategies to improve adherence to interventions {11c}

All therapy sessions will be digitally recorded, and adherence to treatment procedures will be evaluated using the Cognitive Therapy Rating Scale (CTRS) (Young JE, Beck AT: Cognitive therapy scale, Unpublished manuscript. Yurica CL: Inventory of cognitive distortions: Development and validation of a psychometric test for the measurement of cognitive distortions, Doctoral dissertation) and SBOA rating scale by PI Slesnick. A random sample of 20% of sessions will be coded to ensure that the procedures are delivered as intended.

### Relevant concomitant care permitted or prohibited during the trial {11d}

This trial seeks to link YEH to concomitant care, so no care is prohibited.

### Provisions for post-trial care {30}

The trial seeks to link youth to ongoing care that extends beyond trial completion. No provisions are provided once the trial ends.

### Outcomes {12}

Each of the measures below is assessed at baseline, 3, 6, 9, and 12 months in order to determine change over time (Table 1).

**Table 1:** Suicide prevention protocol schedule of procedures and assessments

Procedures/assessments	Study period					
	**Allocation**	**Baseline**	**Follow-up**			
			**3 months**	**6 months**	**9 months**	**12 months**
Enrolment:						
Eligibility screen	5 min	X				
Informed consent	5 min	X				
Interventions:						
Service as Usual (SAU)	6 months	X	X	X		
Cognitive Therapy for Suicide Prevention (CTSP) + SAU	6 months	X	X	X		
Assessments						
Locator form	5 min	X	X	X	X	X
Demographic/Homeless Experiences Form (HEF)	10 min	X				
Follow-up Homeless Experiences Form (HEF)	10 min		X	X	X	X
Services contact log	5 min	X	X	X	X	X
The Client Satisfaction Questionnaire (CSQ-8)	10 min	X	X	X	X	X
Scale for Suicide Ideation – Worst (SSI-W)	10 min	X	X	X	X	X
Form 90	20 min	X	X	X	X	X
The Beck Depression Inventory II (BDI-II)	10 min	X	X	X	X	X
Inventory of cognitive distortions	5 min	X	X	X	X	X
The Social Problem-Solving Inventory-revised Short-version (SPSI-R:S)	5 min	X	X	X	X	X
The Coping Inventory for Stressful Situations-Short Form (CISS-SFC)	5 min	X	X	X	X	X
The Distress Tolerance Scale (DTS)	10 min	X	X	X	X	X
The Impulsivity Control Scale (ICS)	5 min	X	X	X	X	X
The Social Network Inventory (SNI)	10 min	X	X	X	X	X
The Interpersonal Needs Questionnaire (INQ)	10 min	X	X	X	X	X
Beck Hopelessness Scale (BHS)	10 min	X	X	X	X	X
Acquired Capability for Suicide Scale (ACSS)	10 min	X	X	X	X	X
WAI (Monthly-5 times)	5 min		X	X		
Cortisol levels	10 min	X	X	X	X	X

#### Primary outcome

- Suicide Ideation – Worst total score (SSI-W) [49]

#### Secondary outcomes

- Percentage of days alcohol and drug use (Form-90) [50]
- Depressive symptoms total score from the Beck Depression Inventory II (BDI-II) [51]

#### Primary mediator

- Total cognitive distortions score—Inventory of Cognitive Distortions (ICD) [52]

#### Mediating protective factors

- Total score from the Social Problem-Solving Inventory-revised Short-version (SPSI-R:S) [53]

#### Mediating risk factors

- Total hopelessness scale score (BHS) [54]
- Total score of suicidal capacity (Acquired Capacity for Suicide Scale (ACSS) [22]
- Mean frequency of contact across all network members from the Social Network Inventory (SNI) [55]
- Thwarted belongingness subscale score—the Interpersonal Needs Questionnaire (INQ) [22
- Total distress tolerance score (DTS) [56]
- Physiologic stress is measured through cumulative cortisol levels collected and assayed from hair (each 1 cm of hair growth approximates the cumulative cortisol level for the corresponding month)
- Total coping with stress score from the Coping Inventory for Stressful Situations-Short Form (CISS-SFC) [57]
- Total impulsivity control score (ICS) [58]

#### Moderating variables

- Sex at birth, race/ethnicity, sexual orientation (demographic questionnaire), and baseline service connection (services contact log).

### Participant timeline {13}

Participant flow is shown in Table 2. Eight youth will be recruited each month starting in October, 2023, until the entire sample, *N* = 300, is recruited. Study completion occurs 1 year from the last baseline assessment, and youth are assessed at 3, 6, 9, and 12 months post-baseline.

**Table 2:** Recruitment and assessments by project year

Year	1	2	3	4	5	Total
Baseline assessments	72	96	96	36	0	300
3-month follow-ups	48	96	96	60	0	300
6-month follow-ups	24	96	96	84	0	300
9-month follow-ups	0	96	96	96	12	300
12-month follow-ups	0	72	96	96	36	300
Total assessment interviews	144	456	480	372	48	1500

### Sample size {14}

The power analyses were conducted using the Monte Carlo simulation method [59]. For the LGMs proposed under Aim 1, previous intervention work established a medium to large effect size for treatment differences on suicidal ideation, with *r*s ranging from 0.42 to 0.77 [60] and Cohen’s *d* ranging from 0.64 to 1.03 [61] from 3- to 12-month post-baseline, favoring the cognitive behavioral treatment. Similarly, treatment differences on secondary outcome, depressive symptoms, also have shown medium to large effect sizes, with *d* ranging from 0.39 to 1.24 for depressive symptoms across 6 months [62]. Aim 1 analyses will compare the growth curves of CTSP + SAU to SAU alone. The proposed sample size is 300, assuming an attrition of 10, 15, 15, and 20% at the 3-, 6-, 9-, and 12-month follow-up (Table 2). For the LGMs, under the conditions of continuous outcome variables (e.g., suicidal ideation) and a dichotomous covariate (i.e., two intervention conditions) that has a regression coefficient of 0.20 (medium effect size) for the slope growth factor [59], a sample size of 300 can produce a power of 0.98 to detect the treatment effect on the growth rate of the outcomes. For Aim 2, mediation analyses (path models) are proposed to identify the mechanisms that underlie the treatment effects. Previous clinical trials have reported treatment differences of medium to large (*d* = 0.49 to 0.88) effect sizes for the proposed mediators, such as perceived burdensomeness [61]. Following the three-path mediation model specification suggested by Thoemmes et al. [63], and assuming medium effect sizes for the path coefficients, the proposed sample size can provide sufficient power (0.92) to detect mediation effects.

### Recruitment {15}

Identifying those at risk of suicide is a core objective in national strategies to prevent suicide. Unfortunately, YEH have limited access to medical care and mental health services and are thus unlikely to be screened for suicide in traditional settings. More efforts are needed to identify YEH at risk for suicide and link them to appropriate resources. Therefore, in this study, 50% of the sample will be engaged from the local drop-in center, and 50% of the sample will be non-service-connected recruited through outreach in non-service locations. The outreach worker will engage and screen youths to determine basic eligibility for the study. Those engaged on the streets who meet preliminary eligibility will be transported to the drop-in center. The interviewer will review the nature and conditions of the study, and the informed consent/assent. After signing the consent/assent statement, the interviewer will administer the SSI-W instrument to determine formal eligibility. Those meeting the criteria for participation in the study (score 5 or higher at the SSI-W instrument) will continue with the study. Those not passing inclusion criteria for the project will be provided a care package (with toiletries and food items) and told that even though they are not eligible to participate in the current study, they can continue to receive services through the drop-in center. Those determined to be at imminent risk for suicide will be accompanied to the local emergency room. They can be eligible for the project when they indicate that they are no longer at imminent risk for self-harm.

## Assignment of interventions: allocation

### Sequence generation {16a}

At the end of the baseline assessment, we will use an urn randomization computerized program to ensure that the two study conditions are balanced by baseline service connection and other moderating variables (sex at birth, race, identify as sexual minority).

### Concealment mechanism {16b}

Upon completion of the baseline assessment, the RA contacts the PI with randomization information, and the PI randomizes the client using the computerized urn randomization program.

### Implementation {16c}

RAs enroll participants and the PI assigns each participant to a condition based upon the urn randomization program results.

## Assignment of interventions: blinding

### Who will be blinded {17a}

There is no blinding.

### Procedure for unblinding if needed {17b}

Not applicable as no blinding was used in this trial.

## Data collection and management

### Plans for assessment and collection of outcomes {18a}

Participant data will be collected using REDCap, a secure web application for building and managing online surveys and databases. It is specifically geared to support online or offline data capture for research studies and operations. In order to ensure data quality, all RAs will receive intensive training including videos, in-person trainings, practice, and observation in the field. During the trial, RAs will receive regular supervisions (bi-weekly) and refresher trainings. All assessments will receive quality assurance checks. Outcomes and mediators will be assessed using the following measures.

#### Outcome measures

The 19-item Scale for Suicide Ideation – Worst (SSI-W) is an interviewer-administered rating scale [49], which will be used as the measure of the study’s primary outcome as well as to evaluate youth’s eligibility for the study. The SSI-W was adapted from the Scale for Suicide Ideation (SSI)—one of the most widely used instruments developed for rating suicidal ideation to identify the intensity of the most severe suicidal ideation experienced by the person. In this trial, the same questions will be utilized to rate youth’s most severe suicidal ideation during the prior 90 days. The SSI-W has moderately high internal consistency (*α* = 0.88). The scale also has established validity, showing significant associations with other measures of suicidal ideation including the SSI, the suicide item from the Beck Depression Inventory, and the suicide item from the Hamilton Rating Scale for Depression [51]. The primary measure of substance use quantity and frequency will be the interviewer-administered Form 90 Substance Use Interview [50]. This interview yields total number of days, in 90 days prior to last use, of all alcohol/drug use, total number of drugs used, age at first use, lifetime weeks of use, and level of use. This measure also assesses housing, education, and employment days. The Beck Depression Inventory II [51] is the most frequently used self-report instrument for assessment of mood, and cognitive and somatic aspects of depression and has been used with adults and adolescents. The BDI’s internal consistency estimates yielded a mean coefficient alpha of 0.86 for psychiatric patients. The mean correlations of the BDI samples with clinical ratings and the Hamilton Psychiatric Rating Scale were 0.60 and 0.74, respectively [51].

#### Primary mediator

The Inventory of Cognitive Distortions (ICD) is a 69-item self-report questionnaire designed to assess cognitive distortions in clinical populations [52]. The ICD contains 11 scales, each assessing a distinct cognitive distortion. Each item is rated on a Likert scale from 1 (“never”) to 5 (“always”) and has demonstrated excellent internal consistency reliability (*α* = 0.96). The ICD has also demonstrated strong concurrent validity with measures of dysfunctional attitudes and correlates positively with measures of depression and anxiety [52].

#### Secondary mediators: risk and protective factors

The Social Problem-Solving Inventory-revised Short-version (SPSI-R:S) [53] is one of the most widely used self-report instruments for measuring personal perceptions of social problem-solving ability as it relates to suicidal behaviors. This 25-item assesses both constructive/adaptive and dysfunctional problem-solving dimensions. Strong internal consistency and test–retest reliability (Cronbach’s *α* > 0.90 and *r* = 0.91) and concurrent validity with other measures of social problem-solving and depressive symptomatology have been found [64]. The Coping Inventory for Stressful Situations-Short Form (CISS-SFC) assesses stress coping and includes 21-item measuring that yields 3 subscale scores: task-, emotion-, and avoidance-oriented coping [57]. Reliability for the three subscales is 0.90, 0.88, and 0.83, respectively [57]. Using the Physiologic Stress Measure, we measure chronic physiologic stress of youth through cumulative cortisol levels collected and assayed from hair (each 1 cm of hair growth approximates the cumulative cortisol level for the corresponding month). A recent study assessed the validity of the measure and found cortisol levels assayed in 1 cm of hair were correlated (*r* = 0.61, *p* < 0.01) with salivary cortisol measures in which saliva was collected at 3 time points each day for 1 month [65]. To collect the hair samples, approximately 10–75 mg (approximate width of shoelace tip when bunched) of hair [66] is cut with professional shears from the posterior vertex region of the scalp as close to the scalp as possible. The posterior vertex has the lowest variation in cortisol levels and is the preferred area for sampling [66]. Participants will be surveyed on corticosteroid use as these medications may suppress cortisol levels, psychiatric medication use (e.g., antidepressants), and their hair care practices. Hair will be assayed for cortisol level. The Distress Tolerance Scale (DTS) is a 15-item self-report questionnaire examining the degree to which individuals experience negative emotions as intolerable [56]. The DTS demonstrated high internal consistency and concurrent validity [56]. The Impulsivity Control Scale (ICS) was designed to assess the tendency to engage in impulsive behaviors and lack of patience [58]. The internal reliability for the ICS is 0.80, and the total score was found to be significantly associated with suicidality (*r* = 0.43) and aggressiveness (*r* = 0.63) [58]. The Social Network Inventory (SNI) is a modified version of the Network Interview [55]. The SNI has been used in multiple studies with homeless populations and high-risk adolescents. Respondents are asked to answer various questions about people who are important to them, including family members and friends, and with whom they have interacted within the last 6 months. This study will utilize a support contact measure based on the mean frequency of contact across all network members who the respondent indicated as having provided emotional, tangible, or other support. Possible answers range from 0 to 5 with 0 indicating no contact/support in the past 6 months. This instrument has shown test–retest reliabilities of 0.74 to 0.82 for the key SNI variables for homeless populations [67]. The Interpersonal Needs Questionnaire (INQ) is a 25-item self-report scale designed to assess the two components of suicidal desire as conceptualized by the Interpersonal Psychological Theory of Suicide: thwarted belongingness and perceived burdensomeness [22]. The instrument has demonstrated high internal consistency with alpha coefficients ranging from 0.85 to 0.89 [22]. The Beck Hopelessness Scale (BHS) is a self-report instrument that consists of 20 true–false statements designed to assess the extent of positive and negative beliefs about the future during the past week [54]. The BHS is one of the most widely used measures of hopelessness and has demonstrated high internal reliability across diverse clinical and nonclinical populations with Kuder-Richardson reliabilities ranging from 0.87 to 0.93 [54]. The Acquired Capability for Suicide Scale (ACSS) is a 20-item measure [22], based upon the Interpersonal Psychological Theory of Suicide [21], designed to assess fearlessness about lethal self-injury in both clinical and nonclinical samples. The reliability for the ACSS is adequate (*α* = 0.67) [22]. The ACSS total score is strongly correlated with one item from the Beck Scale for Suicide Ideation (BSS) that asks about one’s courage to kill oneself (*r* = 0.79, *p* = 0.007).

### Plans to promote participant retention and complete follow-up {18b}

One concern when working with YEH is the critical issue of tracking youth for follow-up assessments. Smart and Ogborne [68] in working with YEH suggest the use of outreach workers to maintain contact with those who are lost to follow-up. This proposed project will also utilize the efforts of outreach workers. Extensive locator information will be obtained at the baseline assessment in which youth designate hangout spots in Columbus, as well as collaterals. Each month, the outreach worker will review and update the contact and collateral information which the youth provided. Finally, we have found that YEH who feel connected to staff stay in contact. Also, youth are offered $5 food gift card incentive for attending the intervention sessions.

### Data management {19}

RAs will enter questionnaire data into the RedCap electronic data capture tools via laptop or tablet. The files are saved in a secured fire wall protected server. Any paper records will be entered into RedCap. Data quality will be assessed through range checks for data values. Data will be transferred to a statistical program for analyses.

### Confidentiality {27}

Federal guidelines will be followed regarding the protection of subjects in alcohol/drug studies including using protections offered by the Certificate of Confidentiality. Subjects will be informed that paper records will be kept in a locked and secure records area in the drop-in center offices and transferred to the Ohio State University where they will also be stored in a locked and secure records area. Anonymity will be maintained by labeling materials with identification numbers instead of names.

Electronic data is firewall protected by a Cisco PIX Security Appliance. Cisco’s Adaptive Security Algorithm provides stateful packet inspection firewall services. Authorized network communications are tracked and unauthorized attempts are blocked. The PIX uses the Syslog service to log both inbound and outbound traffic to a Syslog server. Daily logs are monitored daily and stored monthly. All users have passwords that are re-generated and changed every 180 days. Individual files are further protected by user/owner set protection. Only certain users have write privileges on these databases. Backups are run daily on volatile datasets. System-wide weekly and monthly backups are performed.

### Plans for collection, laboratory evaluation, and storage of biological specimens for genetic or molecular analysis in this trial/future use {33}

To collect the hair samples, approximately 10–75 mg (approximate width of shoelace tip when bunched) of hair [65, 66] is cut with professional shears from the posterior vertex region of the scalp as close to the scalp as possible. The posterior vertex has the lowest variation in cortisol levels and is the preferred area for sampling [66]. Hair will be assayed for cortisol at the Ford (Co-I) lab at the Ohio State University.

## Statistical methods

### Statistical methods for primary and secondary outcomes {20a}

Latent growth models (LGM) and mediational models, as described below, will be estimated using MPlus 8 program and evaluated using generally accepted criteria including chi-square, RMSEA, CFI, and SRMR model fit statistics.

#### Study aim 1

The impact of the two study conditions (i.e., CTSP + SAU and SAU alone) on the outcomes will be tested in Aim 1. For suicidal ideation, a latent growth model (LGM) will be applied to the longitudinal data across 5 time points (baseline, 3, 6, 9, 12 months) to estimate the trajectories of change in suicidal ideation over time. Treatment effects on estimated growth parameters, including the intercept (the initial status) and the slope (trends of change over time), will be specifically tested. It is expected that those assigned to CTSP + SAU will show a sharper decrease in suicidal ideation and maintain a lower level following the treatment than those assigned to SAU alone. Similar LGM and follow-up analyses will be conducted on the secondary outcomes, substance use frequency, and depressive symptoms.

#### Study aim 2

To determine the change mechanisms underlying the treatment effects, a series of mediation models will be estimated. Hypothesis 2a tests whether changes in youths’ cognitive distortions will mediate treatment differences on the outcomes. We expect that CTSP will lead to a decrease in cognitive distortions which will predict reduced suicidal ideation (primary outcome) and reduced substance use and depressive symptoms (secondary outcome). Hypothesis 2b tests the proposed risk and protective factors on the association between cognitive distortion and suicidal ideation as well on secondary outcomes. We expect that lower cognitive distortion at the 3-month follow-up will be associated with higher levels of protective factors and lower levels of risk factors at the 6-month follow-up, which in turn will be related to lower suicidal ideation and secondary outcomes at the 12-month follow-up. In these models, the baseline assessment of the mediators and outcomes will be controlled in the analysis. The product of the coefficient of the path from the independent variable to the primary mediator (a), the coefficient of the path from the primary mediator to the secondary mediator (b_1_), and the coefficient of the path from the secondary mediator to the outcomes (b_2_) will be computed as the indirect (mediation) effect between the treatment and outcomes (ab_1_b_2_). The strength and significance of the mediation will be estimated using a bootstrap sampling method. The mediation model for each outcome variable (primary and secondary) will be tested separately.

### Interim analyses {21b}

No interim analyses are planned.

### Methods for additional analyses (e.g., subgroup analyses} {20b}

#### Outreach worker effects

To ensure that differences between outreach workers are not affecting improvements in main variables of interest and engagement into treatment, analyses between them will be conducted prior to the planned statistical tests. Using outreach worker as the independent variable and number of completed sessions and suicidal ideation at the 6-month follow-up (end of treatment) as the dependent variables, an analysis of variance will be conducted to examine differences. If there is a significant effect, then outreach worker will be used as a control variable in the planned latent growth models and mediation models. Otherwise, outreach workers will not be controlled in the analyses.

#### Study aim 3. Moderating effects

We will explore the effect of moderating variables (sex, race, sexual orientation, baseline service connection) by examining the interaction between the intervention outcomes and each moderator in predicting outcome slopes in the growth models. The contrast between intervention outcomes will be multiplied by each moderator variable to form an interaction term. Next, the interaction terms will be included in the analyses for Aim 1 for the primary outcome, one at a time, and their strength of association with the outcome slope will be assessed while controlling the direct effects of the predictors on outcome and the direct effect of moderator variables on outcome. If the interaction term is significant, this suggests moderation.

#### Study aim 4. Rigorous assessment of acceptability, feasibility, fidelity, and cost of CTSP for future dissemination efforts

We will use a sequential mixed-methods design as described below.

##### Quantitative data analysis

To examine youths’ perceptions of CTSP, descriptive statistics will be used to summarize indicators of fidelity, acceptability, feasibility, and cost. To examine *fidelity*, descriptive statistics will summarize the fidelity coding instrument (CTRS) scores. We will also explore *acceptability* through youth’s satisfaction scores (CSQ) and whether perceptions differ based on service-connection or demographics. Assessment of *feasibility* includes documentation of (1) whether participants were recruited and maintained in the prevention services as proposed, data which can be obtained from RA screening forms and session records (meetings attended), and (2) whether the recruitment timeline proposed, and other intervention procedures can be maintained as proposed. Average start-up and ongoing estimated *cost* of delivering CTSP will be presented.

##### Qualitative analysis

Transcripts will be analyzed iteratively by two independent coders (Drs. Chavez and Brakenhoff) using a modified grounded theory approach—modified in that we will orient our initial open coding process around what Patton describes as “sensitizing concepts” including issues related to acceptability, appropriateness, and feasibility [69]. NVivo 11 software will be used to organize and code the data.

##### Integrating qualitative and quantitative data analysis

Quantitative findings may be used to inform some of the qualitative data collection. For example, if some youth are found to be less likely to engage in the intervention or services, we will use the qualitative interview as an opportunity to ask youth about their perception of factors that influenced their participation. Data will be used to identify salient contextual features of service delivery that need to be addressed in future implementation efforts.

### Methods in analysis to handle protocol non-adherence and any statistical methods to handle missing data {20c}

#### Follow-up attrition

Potential biases introduced by differential rates of follow-up by study group will be assessed using 2 × 2 chi-square tests at the 3-, 6-, 9-, and 12-month follow-ups (treatment group, 2 levels, attrition yes/no, 2 levels). Systematic attrition related to participant baseline characteristics (e.g., problem severity) also possesses threats to the internal and external validity of the study. Logistic regression will determine if measures of client functioning at baseline predict follow-up status (e.g., lost).

#### Missing data

Missing data will be examined to determine if data are missing completely at random (MCAR) or missing at random (MAR). Full information maximum likelihood (FIML) or the multiple imputation (MI) method will be used to estimate missingness and are shown to produce unbiased results. MPlus will be used as it can handle missing data by providing FIML estimation; otherwise, the pattern mixture model framework will be used to conduct data analyses.

### Plans to give access to the full protocol, participant-level data, and statistical code {31c}

Data from surveys will be deposited and made available through the Inter-University Consortium for Political and Social Research (ICPSR), which is an NIH-funded repository 1 year from project completion. These data will be shared with investigators working under an institution with a Federal Wide Assurance (FWA) and could be used for secondary study purposes.

## Oversight and monitoring

### Composition of the coordinating center and trial steering committee {5d}

This is a single-site study, so there will be no coordinating between centers. This trial does not have trial steering committee. However, the PI will meet regularly with the management team, co-investigators, and data and safety monitoring board.

### Composition of the data monitoring committee, its role and reporting structure {21a}

A board comprised of three members experienced in clinical trials and working with vulnerable populations will be convened twice yearly to review safety and progress. Adverse events will be monitored, and whether youths show deterioration as a function of the intervention received will be assessed (e.g., increase in substance use, mental health symptoms). The board will prepare a summary report after each meeting. The report will detail the meeting proceedings and recommend changes to study protocol, if deemed necessary. The board’s summary report and other required data reports on adverse event cases will be submitted to the IRB and our NIDA project officer.

### Adverse event reporting and harms {22}

Adverse events are defined as an event that has not been previously observed or is not consistent in nature, severity, or frequency with existing risk information. Serious adverse events are defined as an adverse event that is fatal or life threatening, permanently disabling, requires or prolongs hospitalization, or results in significant disability, congenital anomaly, or birth defect. Should a client experience a serious adverse event that was unanticipated and believed to be related to study procedures, the OSU IRB and NIDA will be notified within 48 h. Adverse events that are unexpected and related to the study, but not meeting the definition of a serious adverse event, will be reported to the IRB within 10 days of the PI’s discovery of the event. The OSU IRB reviews the adverse event report and determines if the event is a result of study procedures. If the event is considered a direct result of study procedures, the PI and the board will meet within 48 h and will discuss the modifications to the protocol that are needed in order to prevent future adverse events. An adverse event report will be distributed to all board members. After the session, the board will send a summary report to all of the project investigators outlining the board’s recommendations for necessary changes to the protocol based on the adverse event which will then be forwarded to the IRB.

### Frequency and plans for auditing trial conduct {23}

The DSMB will meet to review trial conduct every 6 months.

### Plans for communicating important protocol amendments to relevant parties (e.g., trial participants, ethical committees) {25}

Significant amendments to the protocol will be approved by IRB and reported to the federal sponsor.

### Dissemination plans {31a}

The trial is detailed in ClinicalTrials.gov. Study findings will be presented at national and/or international conferences and to local, community outlets. All final peer-reviewed manuscripts will be submitted to the digital archive, PubMed Central. The findings of the trials will be summarized and posted on the lab’s website, so it can be accessed by public.

## Discussion

Much of the literature focuses on characterizing the predictors of suicidal ideation and suicide, but less research has focused on testing prevention interventions, especially with high-risk, marginalized substance using youth, and none with YEH. Although not all individuals who have suicidal ideation die by suicide, suicidal ideation is a central component of suicidal acts and researchers have demonstrated that suicidal ideation is a robust predictor of suicide attempts and deaths. Our ultimate interest is prevention of lethal suicide, but this assessment is typically conducted over a period of years through examination of public records [70]. Thus, by targeting suicidal ideation, this study adds to a very small number of clinical trials seeking to prevent suicide among a very high-risk group by addressing suicidal ideation directly rather than underlying conditions. The study design uniquely recruits service disconnected youth, estimated to represent 90% of those at risk. We assert that community-based outreach and engagement may be essential to effectively intervene in YEH at risk for suicide, reducing disparities in service access. Our overall study methodology includes several infrequently used procedures. First, we will employ outreach workers as the providers, increasing the potential reach and uptake of the needed mental health intervention. Second, physiological measures of stress associated with physical and mental health have primarily been obtained using cross-sectional rather than longitudinal designs [71]. Finally, we include a rigorous assessment of cost, fidelity, acceptability, and feasibility of delivery to inform future implementation efforts by drop-in centers and shelters around the country. To our knowledge, this is the first line of research to test a suicide prevention intervention with substance using YEH.

## Trial status

The ClinicalTrials.gov protocol is Version 1, registered August 8, 2023. Trial recruitment is expected to begin in November 2023, with estimated completion November 2026. Study follow-ups are expected to be completed November 2027. The study number of this trial is 2023B0145, Version 1 approved August 4, 2023.

## Data Availability

Final de-identified datasets will be deposited and made available through Inter-University Consortium for Political and Social Research (ICPSR), which is an NIH-funded repository, and that these data will be shared with investigators working under an institution with a Federal Wide Assurance (FWA) and could be used for secondary study purposes.

## Abbreviations

YEH: Youth experiencing homelessness
CTSP: Cognitive Therapy for Suicide Prevention
SAU: Services as Usual
RCT: Randomized controlled trial
SBOA: Strengths-based Outreach and Advocacy
SSI-W: Scale for Suicide Ideation-worst
WAI: Working alliance inventory
CSQ: Client Satisfaction Questionnaire
BDI-II: The Beck Depression Inventory II
ICD: Inventory of Cognitive Distortions
SPSI-R:S: The Social Problem-Solving Inventory-revised Short-version
CISS-SFC: Coping Inventory for Stressful Situations-Short Form
BHS: Beck Hopelessness Scale
ACSS: Acquired Capability for Suicide Scale
CTRS: Cognitive Therapy Rating Scale
RA: Research assistant
PI: Principal investigator
CFIR: Consolidated Framework for Implementation Research
FIML: Full information maximum likelihood
MI: Multiple imputation
MCAR: Missing completely at random
MAR: Missing at random
MNAR: Missing not at random
LGM: Latent growth models
